# MyPreventiveCare: implementation and dissemination of an interactive preventive health record in three practice-based research networks serving disadvantaged patients—a randomized cluster trial

**DOI:** 10.1186/s13012-014-0181-1

**Published:** 2014-12-11

**Authors:** Alex H Krist, Rebecca A Aycock, Rebecca S Etz, Jennifer E Devoe, Roy T Sabo, Robert Williams, Karen L Stein, Gary Iwamoto, Jon Puro, Jon Deshazo, Paulette Lail Kashiri, Jill Arkind, Crystal Romney, Miria Kano, Christine Nelson, Daniel R Longo, Susan Wolver, Steven H Woolf

**Affiliations:** Department of Family Medicine and Population Health, Virginia Commonwealth University, Richmond, VA, Virginia; OCHIN, Portland, OR, Oregon; Department of Family Medicine, Oregon Health & Science University, Portland, OR, Oregon; Department of Medical Informatics and Clinical Epidemiology, Oregon Health & Science University, Portland, OR, Oregon; Department of Biostatistics, Virginia Commonwealth University, Richmond, VA, Virginia; Department of Family Medicine, University of New Mexico, Albuquerque, NM Mexico; Department of Internal Medicine, University of New Mexico, Albuquerque, NM Mexico; Department of Internal Medicine, Virginia Commonwealth University, Richmond, VA, Virginia; Center on Society and Health, Virginia Commonwealth University, Richmond, VA, Virginia

**Keywords:** Health promotion, Information management, Informatics, Primary health care, Patient-centered care

## Abstract

**Background:**

Evidence-based preventive services for early detection of cancer and other health conditions offer profound health benefits, yet Americans receive only half of indicated services. Policy initiatives promote the adoption of information technologies to engage patients in care. We developed a theory-driven interactive preventive health record (IPHR) to engage patients in health promotion. The model defines five levels of functionality: (1) collecting patient information, (2) integrating with electronic health records (EHRs), (3) translating information into lay language, (4) providing individualized, guideline-based clinical recommendations, and (5) facilitating patient action. It is hypothesized that personal health records (PHRs) with these higher levels of functionality will inform and activate patients in ways that simpler PHRs cannot. However, realizing this vision requires both technological advances and effective implementation based upon clinician and practice engagement.

**Methods/design:**

We are starting a two-phase, mixed-method trial to evaluate whether the IPHR is scalable across a large number of practices and how its uptake differs for minority and disadvantaged patients. In phase 1, 40 practices from three practice-based research networks will be randomized to add IPHR functionality to their PHR versus continue to use their existing PHR. Throughout the study, we will engage intervention practices to locally tailor IPHR content and learn how to integrate new functions into their practice workflow. In phase 2, the IPHR to all nonintervention practices to observe whether the IPHR can be implemented more broadly (Scalability). Phase 1 will feature an implementation assessment in intervention practices, based on the RE-AIM model, to measure Reach (creation of IPHR accounts by patients), Adoption (practice decision to use the IPHR), Implementation (consistency, fidelity, barriers, and facilitators of use), and Maintenance (sustained use). The incremental effect of the IPHR on receipt of cancer screening tests and shared decision-making compared to traditional PHRs will assess Effectiveness. In phase 2, we will assess similar outcomes as phase 1 except for effectiveness.

**Discussion:**

This study will yield information about the effectiveness of new health information technologies designed to actively engage patients in their care as well as information about how to effectively implement and disseminate PHRs by engaging clinicians.

**Trial registration:**

ClinicalTrials.gov: NCT02138448

**Electronic supplementary material:**

The online version of this article (doi:10.1186/s13012-014-0181-1) contains supplementary material, which is available to authorized users.

## Background

Evidence-based preventive services for the early detection of cancer and other health conditions offer profound health benefits, yet Americans receive only half of indicated services [1]. Colorectal, breast, cervical, and prostate cancers are the leading causes of U.S. cancer deaths, claiming 127,860 lives annually [2]-[4]. Though many forms of cancer screening can reduce mortality, a sizable proportion of the eligible population does not receive recommended screening tests. Only 54% of adults are up-to-date with colorectal cancer screening, 74% with breast cancer screening, and 80% with cervical cancer screening [5]. Less than half of adults are up-to-date with clinical preventive services generally [1],[6], and the gap is even more pronounced among low-income Americans and racial and ethnic minorities [7]. A variety of barriers affect patients, clinicians, and health-care systems [8]-[10]. Patients may be unaware that they need screening, lack motivation to be tested, or face logistical challenges. Clinicians may not promote needed tests due to oversight, lack of time, and/or competing demands [11]. The health system is fragmented and often lacks resources, financing, and support to close these gaps. Cancer screening has the added challenge of requiring shared decision-making [12]-[14] and individualized risk assessment [15] to determine at what age to start or stop screening, how often to rescreen, which test to use (e.g., colonoscopy versus stool blood test), or whether to screen at all (e.g., PSA testing) [16]. Confusion is compounded by inconsistencies among guidelines [16]-[20], weak evidence supporting some screening tests [21],[22], and public over-enthusiasm for screening [23].

Some of these problems might be alleviated by health information technology [24], especially personal health records (PHRs). Some PHRs can offer patients direct access to their electronic health record (EHR) [25],[26], which is empowering, speeds access to past screening dates and results, and enables patients to discover potential inaccuracies in their record. The next generation of PHRs could offer even higher functionality [27]—they could be programmed to apply evidence-based guidelines to assess prevention needs and to incorporate personal data to shape individualized recommendations. The modern information age can enable patients to link prevention guidelines with evidence-based educational resources and decision aids, community services, logistical details, and reminders. Automation has the potential to ease the information burden on clinicians and empower patients with better information. Additionally, while early EHR adopters documented that because of the digital divide, at-risk populations less frequently used PHRs [28], the digital divide has been steadily closing—particularly for mobile applications [29]. Many leading disparities experts now embrace technology as having potential to reduce disparities in care [30].

There is, however, a shortage of objective evidence that PHRs can achieve these lofty aims. A minority of patients have a PHR [31], and most PHRs lack the enhanced functionality described above. The PHRs that connect patients with their medical record often fail to explain content in lay language. Some PHRs offer cancer screening recommendations but rely on simple age- and gender-based logic, ignoring other risk factors. There is little empirical evidence about adoption of highly functional PHRs in typical clinical settings, whether a range of patients will use such systems, what functions patients will use, and how use will be incorporated into practice workflow and patient care.

### Improving functionality of patient health records

We propose an innovative solution to the above problems based on a conceptual model that defined five levels of functionality to make information technology patient-centered [27]. These levels include: (1) collecting patient reported information, (2) collecting existing clinical information from EHRs, (3) translating medical information into lay language, (4) providing individualized recommendations by applying information to evidence-based guidelines, and (5) facilitating informed patient action through embedded information resources and tools. We created an innovative application that can be added to existing PHRs, known as an Interactive Preventive Health Record (IPHR), which features these five levels of functionality and is currently integrated into several major EHRs and PHRs (see www.MyPreventiveCare.org) [27],[32]-[34].

In brief, the IPHR addresses 18 clinical preventive services, including screening tests, counseling services, preventive medications, and immunizations recommended by the U.S. Preventive Services Task Force (USPSTF) [35]. Patients are able to access the IPHR through their practice’s PHR, through a single sign-on integration that links the IPHR to the patient’s EHR record—essentially, the IPHR will function as a seamless application within the existing practice PHR. The IPHR extracts hundreds of clinical data elements to individualize preventive recommendations. Patients also complete a health risk assessment to provide information not available in the EHR but necessary for making preventive service recommendations. Based on this information and the USPSTF guidelines, the IPHR applies programmed logic to generate a personally tailored list of recommendations. The interface offers patients’ hyperlinks to detailed personal messages that explain the preventive service and its rationale; relates relevant details in the patient’s history (e.g., prior laboratory test values and dates) to the personalized recommendations; incorporates motivational interviewing content; includes links to evidence-based educational material, decision aids, and local resources based on each patient’s profile; and summarizes the next steps. Message content is modeled after the U.S. Department of Health and Human Services www.Healthfinder.gov and incorporates feedback from patient usability tests, clinician focus groups, and longitudinal patient and clinician advisory boards where stakeholders function as co-investigators to help create and update content [36]. Content can be further tailored to meet individual user’s needs (e.g., low health literacy content, large print, audio, culturally appropriate images, local resources, free or subsidized services, visual displays for mobile devices). After patients use the IPHR, the system automatically forwards a summary to the EHR inbox of the patient’s clinician for appropriate follow-up.

Currently, most commercial PHRs can deliver only the levels 1 and 2 functions (see Table 1) described in our model and sometimes, elements of level 4 (basic age- and gender-based recommendations). However, prior research suggests that the other functions—translating content into lay language (level 3), robust personalized recommendations (level 4), and facilitating informed patient action (level 5)—are essential to promote cancer screening [37]-[48]. Successful implementation in the clinical environment requires local tailoring, redesign of practice systems, and care coordination with the patient’s team of caregivers (i.e., personal clinician, nurses, and support staff). As with any implementation strategy that requires local tailoring, the scalability of implementing such a system must be examined.

**Table 1:** **Model for functionalities of a Patient-Centered Health Information System**

Functionalities	
Level 1 Functionality: patient reported information	Collect information, such as self-reported demographic and risk factor information as well as patient reported outcomes
Level 2 Functionality: existing clinical information	Integrate patient reported information with existing clinical information from electronic health records and/or claims data
Level 3 Functionality: interpretation of information	Interpret information for the patient by translating clinical findings into lay language and delivering health information through a user-friendly interface
Level 4 Functionality: individualization of information	Provide individualized recommendations to the patient, such as screening reminders, based on the patient’s risk profile and on evidence-based guidelines
Level 5 Functionality: patient activation and engagement	Facilitated informed patient action integrated with primary and specialty care through the provision of vetted health information resources, decision aids, risk calculators, personalized motivational messages, and logistical support for appointments and follow-up

LEGEND. Adapted from Krist AH, Woolf SH. A vision for Patient-Centered Heath Information Systems. *JAMA* 2011; 305(3):300-301.

### Preliminary studies

Three previous studies have evaluated the IPHR’s feasibility. The first was a randomized controlled trial to test whether mailing patients an invitation to use the IPHR increased delivery of services (R18 HS17046-01, Efficacy Trial, 2007–2010) [34]. Two prospective, observational time-series analyses expanded IPHR implementation to an entire primary care practice population in eight practices (R21 HS018811-01, Adoption Trial, 2010–2012) and to six additional practices in Virginia (RFTO #17 290-07-100113, Implementation Trial, 2009–2011) [49]. These studies culminated in the production of a *How-to Guide for Using Patient-Centered Personal Health Records for Prevention,* which is being disseminated nationally by the Agency for Healthcare Research and Quality (AHRQ) and which will inform this project’s implementation [50]. Our findings demonstrate that the IPHR is technically feasible, increases delivery of preventive services for patients enrolled in a controlled trial, and can be fielded to an entire primary care population. However, these tests occurred in a small group of Virginia practices with a relatively homogenous patient population.

Through these studies, the IPHR was integrated into three EHRs (EpicCare™, Enterprise™, and Professional™) that represent 31% of the U.S. EHR market share [51]. The IPHR has also been integrated into two commercial PHR platforms (MyChart™ and FollowMyHealth™). Currently, more than 70,000 patients and 190 clinicians are using the IPHR. With this integration, practices successfully incorporated the IPHR into their workflow—using it to prepare patients for visits, augment health behavior counseling discussions, explain test results, issue automatic patient reminders for overdue services, prompt clinicians about services patients need during encounters, and formulate personalized prevention plans. However, considerable variation was documented, from 2% to 60%, in the proportion of clinicians’ patients who used the IPHR.

## Methods

**Figure 1:**
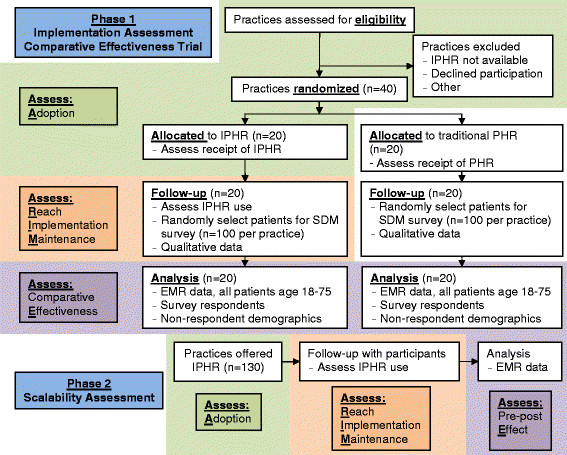
**Study CONSORT flow diagram.**

This study is a two-phase, mixed-method trial designed to assess implementation (phase 1) and scalability (phase 2) with an embedded “real-world” comparative effectiveness trial of a traditional PHR versus a PHR with added IPHR functionality (see Figure 1 for the CONSORT study flow diagram). Implementation will involve both technological and practice-level adaptations, to be studied in three networks spanning eight states. Implementation targets are based on Glasgow’s RE-AIM model [52]-[55]. Adoption, Reach, Implementation, and Maintenance will assess implementation and scalability, while, Effectiveness will assess comparative effectiveness.

In preparation for phase 1, we will develop baseline assessments, recruit and randomize practices, integrate the IPHR into the EHRs and PHRs of study sites, assist with practice training, and support local tailoring of IPHR content. Phase 1 (*Implementation and Comparative Effectiveness Trial*) will include deployment of the IPHR at 20 intervention practices and then contrasting the effectiveness of the IPHR versus traditional PHR at 20 control practices in terms of delivery of recommended screening test and promotion of shared decision-making. Phase 2 (*Scalability Assessment*) will be assessed by examining the three networks’ ability to extend the IPHR to up to 130 nonintervention practices. At all phases, the study will assess disparities in use and outcomes among disadvantaged patients. For the purpose of this study, disadvantaged patients are defined as minority populations (e.g., African-Americans, Hispanics, and Native Americans) and Medicaid beneficiaries (a surrogate for low income). This study has been approved by the VCU Internal Review Board (IRB HM15307) and contains no more than minimal risk to participants. The risks are limited to breaches of privacy and confidentiality.

### Specific aims

*Specific aim 1*. To test the feasibility and scalability of implementing the IPHR in three diverse practice-based research networks with high proportions of minority and underserved patients.

*Phase 1* (*Implementation Assessment*). To measure four implementation metrics in 20 randomly selected practices (intervention sites from the comparative effectiveness trial below):

- *Sub-aim 1a.* The percent of practices within networks approached to participate in the study that agree to and are able to use the IPHR (*Adoption*).
- *Sub-aim 1b*. The percent of practices’ adult patients who make an office visit, use the IPHR, and receive prevention recommendations during the first (*Reach*) 6 months and subsequent 6 months (*practice-level Maintenance*) after adoption.
- *Sub-aim 1c.* The percent of patients who continue to use the IPHR 6 months after initial use (*patient-level Maintenance*).
- *Sub-aim 1d*. The consistency, variation, and fidelity of IPHR delivery across networks, practices, clinicians, and staff; of practice workflow redesigns; and of reported barriers and facilitators to use (*Implementation*).

*Phase 2—sub-aim 1e* (*Scalability Assessment*). To measure Adoption, Reach, Implementation, and Maintenance of the IPHR when the above implementation is replicated in up to 130 practices.

*Specific aim 2* (*Randomized Comparative Effectiveness Trial, Phase 1*)*.* To compare, relative to traditional PHRs, the incremental effectiveness of the additional IPHR features. The trial will test two hypotheses in 20 interventions and 20 control practices. Patients in practices that use the IPHR will receive greater benefit than patients in practices that use a traditional PHR, with even greater benefits among IPHR users than traditional PHR users, including:

- Increased delivery of indicated preventive services (*Hypothesis 1*)*.*
- Improved shared decision-making for cancer screening (*Hypothesis 2*).

*Specific aim 3* (*Disparities Assessment*). To assess whether IPHR use (Reach), preventive service delivery rates (Effectiveness), and perceptions of the technology differ for disadvantaged patients, defined as minorities and Medicaid beneficiaries, in either phase 1 or phase 2.

### Study sample

Practices are being recruited from three practice-based research networks (PBRNs) that span multiple states (Virginia Ambulatory Care Outcomes Research Network—ACORN [56], OCHIN community health information network—OCHIN [57]-[59], Research Involving Outpatient Settings Network—RIOS Net) [60] (see Table 2). These networks offer: (1) offices with a variety of locations, sizes, cultures, and patients representing primary care; (2) a large proportion of disadvantaged patients for testing IPHR feasibility; (3) informatics infrastructure to support IPHR integration; (4) recent introduction of PHRs as a service to patients, providing an optimal window for comparative effectiveness evaluation; and (5) PBRN infrastructure that includes a multi-disciplinary research team, practice liaisons with established practice relationships, expertise recruiting practices, and expertise with engaging stakeholders and fielding research studies.

**Table 2:** **Health system characteristics**

	VCUHS	OCHIN	UNM
Supporting practice-based research network	Virginia Ambulatory Care Outcomes Research Network (ACORN)	Oregon Community Health Information Network (OCHIN)	Research Involving Outpatient Settings Network (RIOS Net)
Number of practices in network	87	200	250 (clinicians)
Number of practices eligible for study	9	200	21
Electronic health record	Cerner	Epic	Cerner
Patient health record	IQHealth	MyChart	IQHealth
Setting	Urban	Urban, sub-urban, and rural	Urban, sub-urban, and rural
Unique patients seen annually	22,554	1,002,794	136,908
Gender			
Female (%)	59	56	55
Ethnicity			
Hispanic (%)	3	28	43
Race			
African-American (%)	57	6	3
Asian (%)	1	13	2
White (%)	39	74	46
Native American (%)	0.1	1.6	8
Payer mix			
Commercial (%)	62	15	28
Medicaid (%)	18	39	22
Medicare (%)	11	6	14
Self pay/indigent (%)	2 / 7	40	19

The *ACORN network* consists of more than 100 primary care practices reflecting the range of primary care in Virginia [56]. The network includes practices from seven health systems; using dozen of EHRs; located in rural, suburban, and urban settings; and with a range of practice organizational and ownership structures. For the purposes of this study, we are focusing on nine practices from the Virginia Commonwealth University Health System (VCUHS), allowing us to integrate the IPHR with one EHR/PHR that is housed on one server. The VCUHS practices are located in inner city Richmond and serve a predominantly African-American population. *RIOS Net* is a voluntary collaboration of clinicians serving southwest New Mexico’s low-income, medically under-served, and culturally diverse communities [60]. Members include 250 primary care clinicians from academic, Indian health, community health, and private clinics. Similar to ACORN, we will focus on recruiting 21 practices from the University of New Mexico health system that utilize the same EHR/PHR. The *OCHIN* is a nonprofit Health Center Controlled Network headquartered in Portland Oregon [61]. It is a collaborative of over 70 primary health-care systems that operate more than 200 clinics across the United States. OCHIN members are community health centers (CHCs), including federally qualified health centers, rural sites, and school-based health centers. All OCHIN practices will be eligible for participation given that they use the same EHR/PHR housed on a central server.

### Intervention and control conditions

Forty practices will be recruited to participate in phase 1, which will be conducted over the span of 1 year. Eligible practices are those who have had an EHR for at least 6 months, currently have a PHR, and agree to participate. The decision to participate will be made by practices and, by extension, their clinicians. A representative from each practice will provide consent to participate through a memorandum of understanding. Randomization will be done by the biostatistician and research coordinator using a modified blocked design. To preserve allocation concealment, practices will be matched into paired clusters based on their associated health system, percent of Medicaid patients, and percent of practice PHR users and then randomized into the intervention and control conditions on a 1:1 ratio.

**Figure 2:**
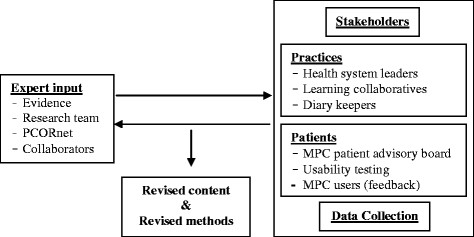
**Engagement of stakeholders.**

The intervention consists of the technical integration of the IPHR into the EHR/PHR, modification of IPHR content to support disadvantaged populations, practice implementation of IPHR functions, and ongoing practice adaptation to use the IPHR. A key element of this study will be to engage intervention practice clinicians, patients, and experts as co-investigators throughout. Stakeholder engagement is depicted in Figure 2. Engagement activities will help to:

- Further inform and refine the study design;
- Advance and locally tailor IPHR content to better meet users’ needs;
- Identify additional PHR functions needed to improve care and decision-making;
- Integrate the IPHR into workflow;
- Interpret findings; and
- Disseminate results locally and nationally.

From a technical perspective, the IPHR will be programmed to integrate with each of the three networks’ EHR/PHR. This will include ensuring that all the required patient data elements for making preventive care recommendations are passed from the EHR to the IPHR, creating links to the IPHR through the practice’s existing PHR, transmitting IPHR clinician summaries and alerts into the practice’s EHR, and locally tailoring IPHR content to meet each practice’s specific resources and needs.

Once technically integrated, all clinicians and staff at intervention sites will be asked to encourage adult patients age 18–75 to use their PHR with its new IPHR features. Three theories will be used to engage the study practices and facilitate cultural and workflow changes necessary to successfully adopt the new IPHR functionality. From Organizational Change Theory, study staff will help practices to: (1) convey a sense of priority for IPHR use, (2) form a guiding coalition, (3) develop and communicate a shared vision, (4) empower practices to act on their vision, (5) plan for short-term wins, and (6) consolidate improvements and institutionalize success [39],[62]-[68]. Organizational Change Theory has been successfully employed for implementing a range of practice changes as well as for adopting the use of health information technology (HIT) [55],[68]-[72]. In addition, drawing from Complex Adaptive Systems Theory, study staff will encourage practices to adopt a variety of solutions, experimenting with and evolving their implementation strategy according to individual practice needs [73]. Finally, using concepts consistent with Diffusion of Innovations Theory, which identifies features that speed adoption, study staff will locally tailor the IPHR in order to ensure that the IPHR is advantageous to practices, provides a better way to deliver prevention, improves quality of care, improves information in the EHR, and is easy to use [74],[75].

To operationalize these theories, practices will be provided with benchmarking, feedback, practice facilitation, online diaries, and learning collaboratives to set priorities, share experiences, and institutionalize successes. Specifically, three groups of practice champions will be assembled, one in each network. Groups will consist of a clinician, nurse, staff, or office manager from each intervention site. Each group will participate in eight learning collaboratives—four sessions in the 4 months prior to adopting the IPHR and four in the year after. Learning collaboratives will be led by the practice liaisons from the PBRNs and follow an agenda successfully used in two prior AHRQ studies [50]. Throughout the process, study staff and champions will collaborate to determine strategies to facilitate implementation. A second site champion will also post biweekly diary entries via email throughout the project. This will be another method to engage stakeholders for input. The research team will read entries in real time, provide feedback to the champion, and discuss message content at research meetings. This methodology was successfully used to evaluate the implementation of 27 interventions in over 200 practices as part of a 5-year RWJF-funded initiative [76],[77].

Practices randomized to the control condition will deliver “usual” cancer screening and “usual” PHR functionality. The networks currently use commercial EHRs/PHRs with levels 1, 2, and (partial) 4 functionality. Control practices will institute ordinary PHR updates. No practices will be in a control condition in phase 2, which follows completion of the randomized trial.

### Data collection

Twelve data sources will be used to address the questions in our three specific aims: EHR data, IPHR data, PHR data, practice records, network records, practice surveys, champion surveys, learning collaborative observations and transcripts, practice recruitment assessments, practice diaries, patient surveys, and patient phone interviews.

#### EHR, IPHR, and PHR databases

The EHR, IPHR, and PHR databases will play a central role in assessing all three aims. Every 6 months during phase 1 and annually during phase 2, network IT staff will transfer EHR data to the research database manager for all patients age 18–75 who make an office visit. Throughout the data transfer process, a standard data transfer protocol will be used that allows us to link patients and their practices across all 12 data sources while maximizing the patients’ privacy and confidentiality. EHR data elements will include patient identification number, age, gender, race-ethnicity, diagnostic codes, family history, orders, screening test dates, and test results. IPHR/PHR data elements will include patient identification number and dates of use. The EHR database will capture data for all patients, irrespective of whether they establish an IPHR or PHR account, whereas the IPHR and PHR databases will contain information only about users.

#### Practice records, network notes, and recruitment assessment

ACORN, OCHIN, and RIOS Net currently collect annual inventories that include basic information about clinicians—e.g., age, gender, degree, years in practice, full-time equivalent—modeled after the Primary Care Network Survey used by AHRQ [78]. Additionally, network PIs and coordinators will complete an assessment when approaching practices to participate in phases 1 and 2 of the study. This information will be used for insights on IPHR adoption and implementation.

#### Practice survey

Practice surveys will be delivered electronically to all practice administrators, clinicians, and staff at intervention practices. Responses will be used as covariates to evaluate Implementation. Surveys will be collected at baseline and 1 year after adopting the IPHR. Questions will address perceptions of preventive care and shared decision-making, perceptions of health information technology, barriers and facilitators to implementing the IPHR, consistency of IPHR implementation, IPHR adaptation over time, and practice culture. We will use questions from (a) the Information Systems Expectations and Experiences (I-SEE) Survey to assess elements of the Technology Acceptance Model (TAM)—a validated pre- and post-implementation survey associated with increased uptake of HIT; [79] (b) an assessment of barriers for adopting HIT; [80] (c) the reciprocal learning scale—an organizational culture characteristic uniquely related to successful HIT implementation and organizational change; [81] and the Patient-Centered Medical Home Assessment (PCMH-A) that address practice workflow [82].

#### Champion survey

A small group of clinicians, nurses, and support staff (2–3 people) from intervention and control practices will complete a baseline workflow and HIT infrastructure assessment. Responses will be used as covariates in our Implementation assessment and responses will be shared with practice champions prior to adopting the IPHR to help inform and guide implementation efforts. The champion survey will include questions from the PCMH-IT and specific questions about preventive care delivery workflow [82].

#### Patient survey

To assess outcomes that cannot be determined from EHR data, surveys will be mailed to 4,000 patients, 100 patients from each of the 40 phase 1 intervention and control practices. A sample of 2,000 patients with office visits in the first 6 months of the trial and 2,000 with office visits in the second 6 months of the trial will be randomly selected to receive surveys. Because the purpose of the study centers on cancer screening, the survey sampling frame will be males age 50–75 and females age 40–75, stratified by age, gender, practice, and PHR use (to include a minimum of 50% PHR users for the subgroup analysis). The modified Dillman method will be used to optimize the response rate for the mail survey [83],[84]. Surveys will be mailed on practice stationery and in practice envelopes and include a $1 incentive [85].

As part of the comparative effectiveness evaluation (outcome #2), surveys will assess components of shared decision-making—knowledge, communication, decisional conflict, and locus of control. Surveys will include a separate set of questions for colorectal, breast, cervical, and prostate cancer screening. National Cancer Institute’s (NCI’s) Health Information National Trends Survey (HINTS) will assess knowledge gained by using the IPHR [86]. Questions will address general knowledge about the cancers, screening tests, screening recommendations, and risks and benefits of screening. To assess whether the IPHR helps patients weigh values regarding risks and benefits, measures of both (a) process (patient-clinician communication) and (b) patient perception (decisional conflict) will be used. Questions from AHRQ’s Consumer Assessment of HealthCare Providers and Systems (CAHPS) survey will be used to measure communication. These questions address the quality of the clinician-patient interaction as well as how often clinicians explained cancer screening, listened carefully, provided understandable instructions, knew the patient’s medical history, and spent enough time with the patient [87]. O’Connor’s low literacy decisional conflict scale will be used to measure patient perspective on whether values were weighed [88]-[90]. Decisional conflict assesses the patient’s uncertainty, how well informed they feel, whether they have clarified their values, and whether they feel supported. To assess whether the IPHR fosters decision-making engagement at the desired level, Degner’s locus of control metric will be used to assess patients’ desired and actual level of involvement in recent cancer screening decisions [91],[92]. The metric will ask patients to choose from one of five options on Degner’s continuum that best describes the relative role they or their clinicians played in making the cancer screening decision.

#### Learning collaborative transcripts and observations

The main purpose of the learning collaboratives, which will occur during the phase 1 implementation assessment, will be to operationalize organizational change theory and engage the practices in creating an IPHR implementation strategy. However, the learning collaboratives will also serve as a rich source of qualitative information to understand the practices’ implementation experience in real time. In addition, learning collaboratives will serve as a setting to engage the clinicians in the development IPHR content and implementation strategies. Practices will identify 1–2 champions per site to participate in the learning collaboratives. Accordingly, we will audio record all meetings, and the study facilitator will take meeting notes to supplement recordings.

#### Practice diaries

The online diaries will be used to see practice implementation through the lens of real-time participants. The entries will identify changes to practice approaches and IPHR content/use that could enhance adoption more broadly. Practices will identify 1–2 diary keepers per site. Diary keepers will be trained on how to use the online diaries during locally based and virtual kickoff meetings in year 1. Diary keepers will be asked to make entries biweekly and share observations and experiences regarding IPHR tailoring, use and adaptation; general and IPHR-specific information about patient engagement in preventive care; and issues related to sustained use of the practice PHR and IPHR. Entries will be read in real time and discussed by the VCU project team. Regular responses will be posted to facilitate rich diary interactions.

#### Patient phone interviews

Interviews with patients will be conducted at the end of phase 1 including 24 IPHR users, 15 who only use the practice PHR, and 9 who do not use the IPHR or the practice PHR. They will be stratified by insurance type (Medicaid versus commercial) and race/ethnicity. The interviews will focus on understanding what made patients use, and continue to use, the IPHR and practice PHR; barriers for engaging online; and effectiveness and perceived value of the IPHR and practice PHR.

### Analytic plan

An overview of our data collection and methods is presented in Table 3. The outcomes for the implementation and scalability assessment are framed around Glasgow’s RE-AIM framework and will include Reach, Adoption, Implementation, and Maintenance [52]-[55]. The comparative effectiveness trial will focus on Effectiveness. All RE-AIM outcome elements will be defined by NCI’s RE-AIM Construct Checklist, with the exception of expenditures [93]. We plan a mixed-method study. The denominator for our *primary intention to treat analysis* of Reach, Effectiveness, and Maintenance will be all patients age 18–75 who are seen for an office visit during the study period. Our access to EHR data in these practices allows us to assemble a complete denominator. We also plan a user *subgroup analysis* to focus on patients in both arms who use the practice’s PHR (IQ Health™ or MyChart™). Given that patients access the IPHR through the practice’s PHR, this subgroup allows us to assess benefit in the subgroup directly exposed to the IPHR. Additionally, this subgroup represents the population most interested in using patient portals—a key target audience for designing more advanced PHRs for the growing number of patients who seek these tools.

**Table 3:** **Overview of data collection methods and analysis**

Aim	Data sources	Analysis
**Aim 1: Implementation:** To field the IPHR and evaluate use in terms of:	•*IPHR/PHR database* to measure which patients use the IPHR or PHR, and when and how often they use it	•Percent of approached practices that agree to use the IPHR **(Adoption)**
		•Percent of patients age 18–75 with a visit who create an IPHR account in months 1–12 **(Reach)** and 13–36 **(practice-level Maintenance)** (monthly repeated measures analysis)
***-Reach***		
	•*EHR database* to measure the number of potential IPHR users (denominator)	
***-*** ***Adoption***		
***-*** ***Implementation***	•*Field notes* to gather quantitative and qualitative insights on practice-level Adoption	•Percent of users who use the IPHR after 6 months **(patient-level Maintenance)**
***-*** ***Maintenance***		•Mixed methods analysis of (quantitative) practice and clinician variation in Reach (two-level mixed-effects logistic regression) and (qualitative) consistency, variation, and fidelity of IPHR delivery (immersion/crystallization analysis of transcripts and diaries) **(Implementation)**
	•*Network records* to measure practice (e.g., size) and clinician characteristics (e.g., age)	
	•*Learning collaborative transcripts*, *practice survey*, *practice diaries*, and *patient interviews* to assess IPHR implementation, including consistency and adaptation, and to qualitatively assess Reach, Effectiveness, and Maintenance	
**Scalability**	Data sources and analysis similar to phase 1 except phase 2 will not include collecting and analyzing learning collaborative transcripts, practice diaries, site visits, or patient interviews	
**Aim 2:** To compare the ***Effectiveness*** of the IPHR vs. traditional PHR functions	•*EHR database* to measure delivery of recommended cancer screening tests	•Percent of patients up-to-date with all indicated cancer screening for all practice patients (intention to treat) and for PHR users (sub-group) (two-level logistic regression)
	•*IPHR/PHR database* to identify users	
		•Shared decision-making outcomes (knowledge, communication, decisional conflict, and decision control) (three-level generalized mixed-effects regression)
	•*Patient survey* of 4,000 randomly selected patients to measure elements of shared decision-making	
		•Patient, practice, and clinician facilitators and barriers associated with Effectiveness (mixed-method analysis)
	•*Learning collaborative transcripts*, *practice survey*, *practice diaries*, and *patient interviews* to explore perceptions	
**Aim 3:** ***Disparities*** **Assessment:** Difference in use, effect and perception of technology for disadvantaged populations	•*EHR database* to identify at risk patients (minorities and Medicaid beneficiaries) and to measure delivery of recommended cancer screening tests	•Comparison of Reach and Effectiveness for the disadvantaged versus general population (two-level mixed-effects logistic regression)
		•Patient interviews to understand technology barriers and needs; technology impact; and unique issues for disadvantaged patients
	•*IPHR/PHR database* to stratify levels of use by minority and Medicaid status	
	•*Patient interviews*	

Italicized words are data collection methods.

Bolded words are specific aim elements that will be assessed.

Whereas the ultimate benefit of the IPHR is to promote recommended preventive services, a secondary benefit of potentially equal importance is to promote shared decision-making. Showing patients their health information, explaining content in lay language, presenting guideline disagreements, and providing decision aids is intended to engage and activate patients to participate in screening decisions. Accordingly, *effectiveness outcomes* include both deliveries of preventive services and elements of the decision-making process defined by Sheridan et al. for the USPSTF (the patient [a] understands the condition, [b] understands the service, [c] weighs values regarding risks and benefits, and [d] is engaged in decision-making at the desired level) [12]. Shared decision-making outcomes to be measured will include patient knowledge, clinician-patient communication, decisional conflict, and difference in desired and actual desired locus of decision-making control.

Our statistical calculations will account for up to three sources of variation: variation within subjects measured repeatedly over time, variation among the physicians who see those patients, and variation among the practices where those physicians see their patients. Note that the variability among physicians will be nested within practices. Patient-level data will be collected for Reach, Effectiveness, and Maintenance, while aggregate outcomes will be used at the practice level. Multi-level, mixed-effect models will be used to account for the above variation in the evaluation of our aims. A similar approach will be used for Adoption and Implementation, although the data collected will be practice-level information.

#### Specific aim 1

*To test the feasibility and scalability of implementing the IPHR in three diverse practice-based research networks with high proportions of minority and underserved patients.* The following strategies will be used for each sub-aim.

1. *Sub-aim 1a. The percent of practices that agree to and are able to use the IPHR* (*Adoption*). Because strategies to field the IPHR will be practice wide, involving a range of staff, Adoption is a practice-level decision. We will use recruitment assessment and network records to calculate descriptive statistics about practices approached, practices willing to use the IPHR, and practices that are able to use the IPHR.
2. *Sub-aim 1b. The percent of patients who use the IPHR in the first (****Reach****) and second and third years* (*practice-level Maintenance*) *after adopting the IPHR.* We define Reach as the percent of patients age 18–75 who make an office visit during the first 6 months after intervention sites adopt the IPHR (denominator) and who (i) sign up for the IPHR, (iii) complete the intake process, and (iii) receive prevention recommendations (each as separate numerators). IPHR data will provide the numerator, and EHR data will provide the denominator. Practice-level Maintenance applies the same definition for patients seen at intervention sites in months 6 through 24 after IPHR adoption.
3. *Sub-aim 1c. The percent of patients who use the IPHR more than 6 months after initial use.* We define *patient-level Maintenance* as the percent of patients who establish an IPHR account (denominator) and revisit the website at least once 6 months after initially establishing their IPHR account (numerator).
4. *Sub-aim 1d. The consistency*, *variation*, *and fidelity of IPHR delivery across networks*, *practices*, *clinicians*, *and staff* (*Implementation*)*.* While it is a practice decision to adopt the IPHR, we expect variation between practices and among clinicians in how they promote and use the IPHR. Quantitatively, we will compare Reach and Effectiveness at the practice-level and clinician-level to quantify variation in implementation at each level, using a two-level mixed-effects logistic regression. From learning collaborative transcripts, practice diaries, and patient interview transcripts, we will use a grounded theory approach and immersion/crystallization techniques to understand how different practices implemented IPHR functions (both initially and over time) and their consistency (versus adaptation) in implementation over time [94]-[100]. Specifically, we will engage project participants in the learning process by sharing emerging insights and having them share in the interpretation of findings. We will use Atlas.ti software to organize, code, and analyze data to identify key findings and themes [101]. The analytic process will include group participation in creating a codebook based on (1) previous experience implementing the IPHR, (2) input and advice from our advisory panel, and (3) key factors that emerge from the data through the immersion-crystallization process of reading and rereading the data, organizing the data around common attributes, and developing an understanding of themes implied by recurrent patterns. Preliminary findings from this analysis will inform phase 2 implementation and scalability.
5. *Sub-aim 1e. Adoption*, *Reach*, *Implementation*, *and Maintenance in the Scalability Assessment.* As the networks disseminate the IPHR across all practices during phase 2, we will measure similar outcomes as described in sub-aims 1a–d, although we will not observe learning collaboratives or hold patient interviews.

#### Specific aim 2

For the two effectiveness hypotheses detailed below, we plan to (a) calculate overall outcomes and stratify outcomes for each specific screening test and (b) compare patients from intervention and control practices for both the intention-to-treat population (all patients age 18–75 seen for a visit) and the user subgroup (all PHR users age 18–75 seen for a visit) as described in the “Specific aims” section. Linear mixed-effect (for numerical outcomes) and generalized linear mixed-effect (for categorical outcomes) models will be used to test the two hypotheses listed below and will account for all three levels of variation (within-subject, physician nested with practice, and practice) using random effects.

1. *Specific aim 2, hypothesis 1. Compare elements of shared decision-making* (*knowledge*, *communication*, *decisional conflict*, *and decision control*) *in intervention versus control practices. Knowledge:* From the patient surveys, we will calculate the percent of correct responses from the series of knowledge questions. We will use a three-level mixed-effects logistic regression for the intention-to-treat and subgroup comparisons. *Process of weighing values* (*communication*): Per the approach defined by CAHPS, we will score the clinician-patient communication questions on a 6-point scale and calculate both the average scores and “top box” scores (percent reporting the most positive responses) [87]. We will use a linear mixed-effect model for the 6-point scale outcome and will use a generalized linear mixed-effect model for the “top box” outcome, in both the intention-to-treat and subgroup comparisons. *Perception of weighing values* (*decisional conflict*): Per protocol, we will score the overall decisional conflict (score 0–100) and the four sub-scores (uncertainty, informed, values, and support) [91],[92]. We will use mixed-effect multinomial logistic regression for the intention-to-treat and subgroup comparisons. *Engagement at the desired level* (*locus of decision control*): Using a mixed-effect multinomial logistic regression, we will compare the proportion of patients expressing differences in preferred and actual locus of decision-making control for intention-to-treat and subgroup comparisons [91],[92].
2. *Specific aim 2, hypothesis 2. Compare the percent of patients who are up-to-date on all indicated cancer screening tests at intervention and control practices*. Our primary Effectiveness outcome for which the study is powered (see below) is the percent of eligible patients who are up-to-date with all indicated cancer screenings (*all-or-none measure*) [102]. Our prior studies demonstrated that the all-or-none measure was the most sensitive to practice-level changes. However, we also plan to measure the percent of indicated cancer screenings that are up-to-date (*composite measure*) and the percent of eligible patients who are up-to-date with each individual cancer screening test [102]. Eligibility for cancer screening and “up-to-date” status will be based on the USPSTF recommendations in effect at the time of the office visit. Prostate cancer screening, breast cancer screening age 40–50, and colorectal cancer screening age 75–85 will be analyzed separately but excluded from the composite and all-or-none measures. We will use a three-level mixed-effect logistic regression for the all-or-none, composite, and individual screening test comparisons.

#### Specific aim 3

*Assess whether the use of the IPHR*, *benefits from the system*, *and perception of the technology differ for disadvantaged patients.* To determine whether exposure to the IPHR affects disparities, we will compare the percentage of minorities and Medicaid beneficiaries versus the general population that use the IPHR (Reach) in both phase 1 (years 2–3) and phase 2 (years 4–5) using a three-level mixed-effect logistic regression. Similarly, we will calculate the difference in delivery of preventive services (Effectiveness), using a three-level mixed-effect logistic regression for the all-or-none, composite, and individual screening tests. Both multi-level analyses will account for practice-level and clinician-level variation. From qualitative material, with an emphasis on the post-implementation patient interviews, we will use a similar grounded theory approach as described in specific aim 1d to understand disadvantaged patients’ perceptions of and experience with the IPHR.

### Sample size

Two power analyses were conducted for the primary (percent of patients up-to-date with recommended cancer screenings) and secondary effectiveness (shared decision-making) outcomes of the study. Data from preliminary studies were used to estimate the anticipated effect size for each outcome. For the primary outcome, it was determined that 40 study sites (20 intervention and 20 control practices) will provide 80% power, alpha = 0.05, to detect an 8% (effect size 0.82) difference in being up-to-date with recommended cancer screening between intervention and control groups in the intention to treat analysis and 80% power, alpha = 0.05, to detect a 15% increase (effect size 0.82) in the subgroup analysis.

For the secondary outcome of the study, it was determined the 100 patients per study site, with stratified sampling to ensure that at least 50% are IPHR users (for the subgroup analysis), will provide 80% power, alpha = 0.05, to detect a 10% knowledge score difference (effect size 0.75–0.80) between IPHR users and nonusers for the intention-to-treat and subgroup analyses, respectively.

### Study status

**Figure 3:**
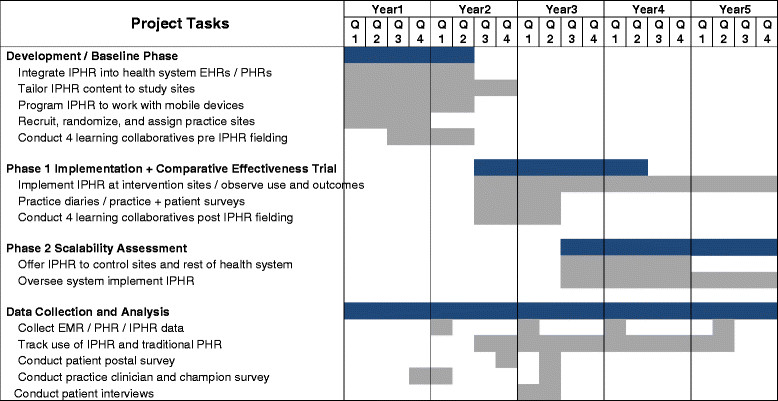
**Study timeline.**

The overall study timeline is shown in Figure 3. Funded in September 2013, the 40 study sites have been recruited from the three PBRNs, practices have been randomized to the intervention and control condition, learning collaborative members and diary keepers have been identified, the first learning collaborative and diary entries have occurred, IPHR is in the process of being updated, and the IPHR is being integrated into the three participating systems’ existing EMR and PHR. We plan to collect baseline data in September 2014, conduct three more learning collaboratives between now and February 2015, and launch the new IPHR functionality 15 February 2015.

## Discussion

The second stage of EHR Meaningful Use will require clinicians to engage 50% of their patients online through PHRs beginning in 2015. It remains to be seen whether clinicians can accomplish this daunting task. It is also unknown whether these efforts will pay off in terms of improved care and health outcomes. We believe that patient-centered PHRs with higher levels of functionality, combined with practice redesign to make use of these functions, can help patients obtain recommended preventive care by linking them to their doctor’s records, explaining information in lay language, displaying tailored recommendations and educational resources, providing logistical support and tools to stimulate action, and generating reminders. Appropriate delivery of these evidence-based services should reduce the burdens of chronic disease and prevent premature death.

This project will measure whether making these resources available to primary care practices and patients promotes shared decision-making and increases the delivery of recommended preventive services compared to existing information systems. Specifically, it will yield needed information about the effectiveness of new health information technologies designed to more actively engage patients in their care as well as information about how to most effectively implement and disseminate PHRs. If the integration of an IPHR into primary care practice is proven effective and scalable, our study findings will have wide ranging practice and policy implications.

We incorporated several important design features to ensure that this study has the potential for maximum impact. For example, the two-phased design allows us to conduct a comparative effectiveness evaluation of current PHR functionality versus embedding more personalized IPHR content followed by a scalability assessment to determine the feasibility and process of broadly integrating IPHR functionality into a wide range of primary care practices. Second, our mixed-method design will provide a robust set of data to understand from multiple perspectives the issues associated with engaging patients in PHR use. This evaluation is proving timely given the recent Stage 2 Meaningful Use requirements. Third, because many of our study practices serve a range of disadvantaged populations, this study will allow us to examine disparities in IPHR and PHR use and how potential disparities in use of these HIT tools might influence outcomes in care. Finally, a core element of our study design is to engage key stakeholders throughout the research project as co-investigators—clinicians, patients, and experts—using a variety of robust methods to ensure that IPHR content meets users’ needs, implementation is integrated into practice workflow, outcomes assessed are meaningful for users, interpretation of findings is guided by users’ experiences, and the voice of our users is incorporated into dissemination activities. Through collaboration with our stakeholders, we hope to ensure our research directions address the prominent concerns of primary care and patients.

Collectively, the above research study design features strike a balance between the fidelity to our interventions that is typically required for internal validity and the need for quality improvement to embrace both individual variation and customization. This National Cancer Institute-funded study is forging the way in striking a balance between these tensions to ensure that our research can be translated to clinical practice in a timely manner.

We recognize that our study has several limitations. While the goal is to improve the delivery of preventive care for all patients, we expect that only a subset of patients will use the IPHR or even the practice’s PHR. We expect that in the future, more and more patients will seek health information online. For the purposes of this study, we are assessing how practices increase their patient’s portal use (Reach). We will also engage patients throughout IPHR refinement to ensure that content is accessible, understandable, and meaningful as well as create mobile ready content that can be accessed on a smartphone. Another study challenge is the broad geographical distribution of our study practices. This makes it more difficult to meaningfully engage local stakeholders, ensure study protocols are carried out as designed, and conduct observational assessments. We plan to use several strategies to mitigate this limitation such as standardizing study materials, using established PBRN infrastructure, building on prior practice relationships, developing methods to promote virtual participation in learning collaboratives (e.g., use of video meetings), and sharing experiences between the three PBRNs.

PHRs hold great potential to improve patient education, promote shared decision-making, facilitate more in-depth conversations, and generally engage patients in their care. However, more is needed from PHRs and from the patients, clinicians, and practices using the PHRs in order to achieve the desired outcomes. This study will evaluate whether making PHRs more patient-centered improves outcomes and it will generate needed evidence about how to engage patients online in primary care.

## Authors’ original submitted files for images

Below are the links to the authors’ original submitted files for images. Authors’ original file for figure 1 Authors’ original file for figure 2 Authors’ original file for figure 3 Authors’ original file for figure 4
