# Supplementary figures and images for: MyPreventiveCare: implementation and dissemination of an interactive preventive health record in three practice-based research networks serving disadvantaged patients—a randomized cluster trial
Source: Implement Sci. 2014 Dec 11;9:181. doi: 10.1186/s13012-014-0181-1 (PMC4269965; doi:10.1186/s13012-014-0181-1)

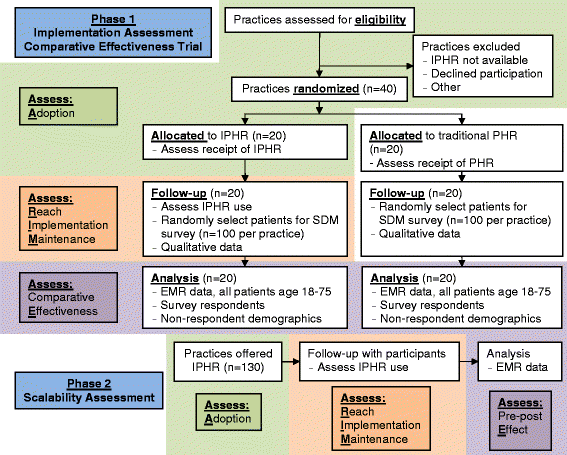

Supplement: Supplementary file 1 — Authors’ original file for figure 1 [file 13012_2014_181_MOESM1_ESM.gif]

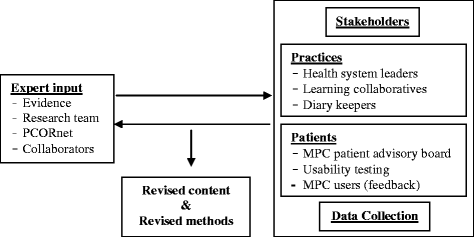

Supplement: Supplementary file 2 — Authors’ original file for figure 2 [file 13012_2014_181_MOESM2_ESM.gif]

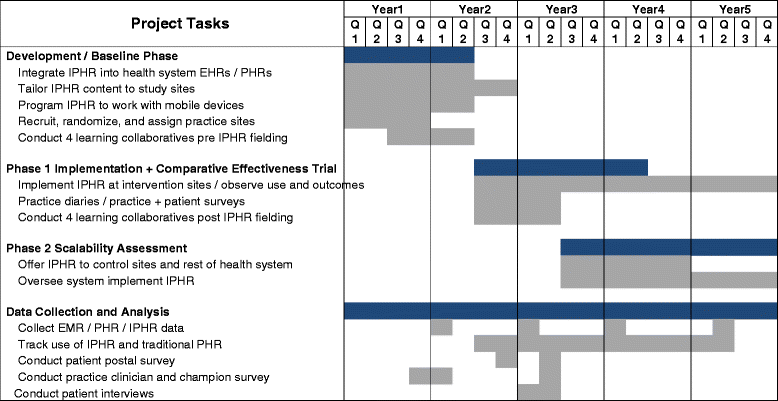

Supplement: Supplementary file 3 — Authors’ original file for figure 3 [file 13012_2014_181_MOESM3_ESM.gif]
