# Supplementary material for: MyPreventiveCare: implementation and dissemination of an interactive preventive health record in three practice-based research networks serving disadvantaged patients—a randomized cluster trial
Source: Implement Sci. 2014 Dec 11;9:181. doi: 10.1186/s13012-014-0181-1 (PMC4269965; doi:10.1186/s13012-014-0181-1)
Supplement: Supplementary file 4 — Authors’ original file for figure 4 [file 13012_2014_181_MOESM4_ESM.docx]

**FIGURE 4. Study Timeline**

| **Project Tasks** | **Year 1** | | | | **Year 2** | | | | **Year 3** | | | | **Year 4** | | | | **Year 5** | | | |
| --- | --- | --- | --- | --- | --- | --- | --- | --- | --- | --- | --- | --- | --- | --- | --- | --- | --- | --- | --- | --- |
|  | **Q 1** | **Q 2** | **Q 3** | **Q 4** | **Q 1** | **Q 2** | **Q 3** | **Q 4** | **Q 1** | **Q 2** | **Q 3** | **Q 4** | **Q 1** | **Q 2** | **Q 3** | **Q 4** | **Q 1** | **Q 2** | **Q 3** | **Q 4** |
| **Development/Baseline Phase** |  |  |  |  |  |  |  |  |  |  |  |  |  |  |  |  |  |  |  |  |
| Integrate IPHR into health system EHRs / PHRs |  |  |  |  |  |  |  |  |  |  |  |  |  |  |  |  |  |  |  |  |
| Tailor IPHR content to study sites |  |  |  |  |  |  |  |  |  |  |  |  |  |  |  |  |  |  |  |  |
| Program IPHR to work with mobile devices |  |  |  |  |  |  |  |  |  |  |  |  |  |  |  |  |  |  |  |  |
| Recruit, randomize, and assign practice sites |  |  |  |  |  |  |  |  |  |  |  |  |  |  |  |  |  |  |  |  |
| Conduct 4 learning collaboratives pre IPHR fielding |  |  |  |  |  |  |  |  |  |  |  |  |  |  |  |  |  |  |  |  |
|  |  |  |  |  |  |  |  |  |  |  |  |  |  |  |  |  |  |  |  |  |
| **Phase 1 Implementation + Comparative Effectiveness Trial** |  |  |  |  |  |  |  |  |  |  |  |  |  |  |  |  |  |  |  |  |
| Implement IPHR at intervention sites / observe use and outcomes |  |  |  |  |  |  |  |  |  |  |  |  |  |  |  |  |  |  |  |  |
| Practice diaries / practice+patient surveys |  |  |  |  |  |  |  |  |  |  |  |  |  |  |  |  |  |  |  |  |
| Conduct 4 learning collaboratives post IPHR fielding |  |  |  |  |  |  |  |  |  |  |  |  |  |  |  |  |  |  |  |  |
|  |  |  |  |  |  |  |  |  |  |  |  |  |  |  |  |  |  |  |  |  |
| **Phase 2 Scalability Assessment** |  |  |  |  |  |  |  |  |  |  |  |  |  |  |  |  |  |  |  |  |
| Offer IPHR to control sites and rest of health system |  |  |  |  |  |  |  |  |  |  |  |  |  |  |  |  |  |  |  |  |
| Oversee system implement IPHR |  |  |  |  |  |  |  |  |  |  |  |  |  |  |  |  |  |  |  |  |
|  |  |  |  |  |  |  |  |  |  |  |  |  |  |  |  |  |  |  |  |  |
| **Data Collection and Analysis** |  |  |  |  |  |  |  |  |  |  |  |  |  |  |  |  |  |  |  |  |
| Collect EMR / PHR / IPHR data |  |  |  |  |  |  |  |  |  |  |  |  |  |  |  |  |  |  |  |  |
| Track use of IPHR and traditional PHR |  |  |  |  |  |  |  |  |  |  |  |  |  |  |  |  |  |  |  |  |
| Conduct patient postal survey |  |  |  |  |  |  |  |  |  |  |  |  |  |  |  |  |  |  |  |  |
| Conduct practice clinician and champion survey |  |  |  |  |  |  |  |  |  |  |  |  |  |  |  |  |  |  |  |  |
| Conduct patient interviews |  |  |  |  |  |  |  |  |  |  |  |  |  |  |  |  |  |  |  |  |
